# Supplementary material for: Emergency care knowledge, utilization, and barriers in Northern Tanzania: A community-based survey
Source: PLOS Glob Public Health. 2025 Jan 6;5(1):e0004032. doi: 10.1371/journal.pgph.0004032 (PMC11703045; doi:10.1371/journal.pgph.0004032)
Supplement: S1 File — (DOCX) [file pgph.0004032.s001.docx]

**Demographics**

1. Survey Number

Namba ya utafiti

1. District

Wilaya

2a. Hai/Hai

2b. Moshi Urban/Moshi Mjini

2c. Moshi Rural/Moshi Vijijini

1. Home address (Or closest description)

Anuani ya makazi (kitu cha karibu kinachoweza kuelezea mahali hapo)

1. Do they live on a paved road?

Je, wenaishi kwenye barabara ya lami?

4a. Yes/Ndio

4b. No/Hapana

1. Can the home be reached by a 4-wheel vehicle or does it need to be reached by foot?

Je, nyumbani kunaweza kufikika kwa gari au kunahitaji kufikika kwa miguu?

5a. Can be reached by car/ kunafikika kwa gari

5b. Can only be reached by foot/ kunafikika kwa miguu tu

5c. Other:/ Nyingine

1. Information provided by: (Name of primary respondent)

Taarifa zimetolewa na: jina la mshiriki

1. Tribe

Kabila

7a. Chagga/ Mchaga

7b. Sukuma/ Msukuma

7c. Muha/ Muha

7d. Sambaa/ Msambaa

7e. Iraqu/ Muiraq

7f. Masai/ Mmasai

7g. Nyaturu/ Mnyaturu

7h. Pare/ Mpare

7i. Mmeru/ Mmeru

7j. Other:/ Nyingine:

1. Religious affiliation

Dini

8a. Christian/Mkristo

8b. Muslim/ Muisilamu

8c. Hindu/ Hindu

8d. Other/None / Nyingine/hakuna

1. Number of Household members

Idadi ya wana kaya

1. Ages and genders of household members

Umri na jinsia za wana kaya

1. Monthly household income

Kipato cha mwezi cha kaya

1. Years of completed education

Miaka ya elimu kwa jumla

1. Level of education completed

Kiwango cha elimu kilichofikiwa

13a. None/ Hakuna

13b. Primary/ Msingi

13c. Secondary/ Sekondari

13d. College/ Chuo

1. Insurance

Bima

14a. None/ Hakuna

14b. Cash personal payment or relative support/ malipo binafsi ya pesa

14c. National Health Insurance/ Bima ya afya ya taifa

14d. Other:/ Nyingine:

1. Medical History

Historia ya matibabu/ugonjwa

15a. Diabetes/ Kisukari

15b. Hypertension/ Shinikizo la damu a juu/presha

15c. Heart disease/ Ugonjwa wa moyo

15d. Stroke/ Kiharusi/stroke

15e. HIV/ VVU

15f. Asthma/ Pumu

15g. Cancer/ Saratani

15h. Any prior surgery/ Upasuaji wowote wa awali

15i. Other/ Nyingine

**HFIAS**

1. In the past four weeks, did you worry that your household would not have enough food?

Katika wiki nne zilizopita, ulikuwa na wasiwasi kwamba kaya yako haitokuwa na chakula cha kutosha?

1a. Yes/ Ndio

1b. No/ Hapana

1b. If yes, how often did this happen?

Kama ndio, Hii ilitokea mara ngapi?

1b.1. Rarely (One or twice in the past four weeks)/ Nadra (mara moja au mara mbili kwa wiki 4)

1b.2. Sometimes (three to ten times in the past four weeks)/ Mara kadhaa (mara 3-10 ndani ya wiki 4)

1b.3. Often (more than ten times in the past four weeks)/ Mara nyingi (Zaidi ya mara 10 ndani ya wiki 4)

1. In the past four weeks, were you or any household member not able to eat the kinds of food you preferred because of a lack of resources?

Katika wiki nne zilizopita, je, Wewe au mtu yeyote wa kaya hakuweza kula aina ya vyakula ulivyopendelea kwa sababu ya ukosefu wa rasilimali?

2a. Yes/ Ndio

2b. No/ Hapana

2b. If yes, how often did this happen?

Kama ndio, Hii ilitokea mara ngapi?

2b.1. Rarely (One or twice in the past four weeks)/ Nadra (mara moja au mara mbili kwa wiki 4)

2b.2. Sometimes (three to ten times in the past four weeks)/ Mara kadhaa (mara 3-10 ndani ya wiki 4)

2b.3. Often (more than ten times in the past four weeks)/ Mara nyingi (Zaidi ya mara 10 ndani ya wiki 4)

1. In the past four weeks, did you or any household member have to eat a limited variety of foods due to a lack of resources?

Katika wiki nne zilizopita, je, Wewe au mtu yeyote wa kaya alilazimika kula aina chache za chakula kwa sababu ya ukosefu wa rasilimali?

3a. Yes/ Ndio

3b. No/ Hapana

3b. If yes, how often did this happen?

Kama ndio, Hii ilitokea mara ngapi?

3b.1. Rarely (One or twice in the past four weeks)/ Nadra (mara moja au mara mbili kwa wiki 4)

3b.2. Sometimes (three to ten times in the past four weeks)/ Mara kadhaa (mara 3-10 ndani ya wiki 4)

3b.3. Often (more than ten times in the past four weeks)/ Mara nyingi (Zaidi ya mara 10 ndani ya wiki 4)

1. In the past four weeks, did you or any household member have to eat some foods that you really did not want to eat because of a lack of resources to obtain other types of food?

Katika wiki nne zilizopita, je! Wewe au mtu yeyote wa kaya alilazimika kula vyakula ambavyo hautaki kula kwa sababu ya ukosefu wa rasilimali kupata chakula cha aina nyingine?

4a. Yes/ Ndio

4b. No/ Hapana

4b. If yes, how often did this happen?

Kama ndio, Hii ilitokea mara ngapi?

4b.1. Rarely (One or twice in the past four weeks)/ Nadra (mara moja au mara mbili kwa wiki 4)

4b.2. Sometimes (three to ten times in the past four weeks)/ Mara kadhaa (mara 3-10 ndani ya wiki 4)

4b.3. Often (more than ten times in the past four weeks)/ Mara nyingi (Zaidi ya mara 10 ndani ya wiki 4)

1. In the past four weeks, did you or any household member have to eat a smaller meal than you needed because there was not enough food?

Katika wiki nne zilizopita, je! Wewe au mtu yeyote wa kaya alilazimika kula chakula kidogo kuliko vile ulivyohisi unahitaji kwa hakukuwa na chakula cha kutosha?

5a. Yes/ Ndio

5b. No/ Hapana

5b. If yes, how often did this happen?

Kama ndio, Hii ilitokea mara ngapi?

5b.1. Rarely (One or twice in the past four weeks)/ Nadra (mara moja au mara mbili kwa wiki 4)

5b.2. Sometimes (three to ten times in the past four weeks)/ Mara kadhaa (mara 3-10 ndani ya wiki 4)

5b.3. Often (more than ten times in the past four weeks)/ Mara nyingi (Zaidi ya mara 10 ndani ya wiki 4)

1. In the past four weeks, did you or any household member have to eat fewer meals in a day because there was not enough to eat?

Katika wiki nne zilizopita, je! Wewe au mtu yeyote wa kaya alilazimika kula milo michache kwa siku sababu hakukuwa na chakula cha kuthosha?

6a. Yes/ Ndio

6b. No/ Hapana

6b. If yes, how often did this happen?

Kama ndio, Hii ilitokea mara ngapi?

6b.1. Rarely (One or twice in the past four weeks)/ Nadra (mara moja au mara mbili kwa wiki 4)

6b.2. Sometimes (three to ten times in the past four weeks)/ Mara kadhaa (mara 3-10 ndani ya wiki 4)

6b.3. Often (more than ten times in the past four weeks)/ Mara nyingi (Zaidi ya mara 10 ndani ya wiki 4)

1. In the past four weeks, was there ever no food to eat of any kind in your household because of lack of resources to get food?

Katika wiki nne zilizopita, je! Mewahi kutokea kukawa hakukuwa na chakula cha aina yoyote katika kaya yako kwa sababu ya ukosefu wa rasilimali kupata chakula?

7a. Yes/ Ndio

7b. No/ Hapana

7b. If yes, how often did this happen?

Kama ndio, Hii ilitokea mara ngapi?

7b.1. Rarely (One or twice in the past four weeks)/ Nadra (mara moja au mara mbili kwa wiki 4)

7b.2. Sometimes (three to ten times in the past four weeks)/ Mara kadhaa (mara 3-10 ndani ya wiki 4)

7b.3. Often (more than ten times in the past four weeks)/ Mara nyingi (Zaidi ya mara 10 ndani ya wiki 4)

1. In the past four weeks, did you or any household member go to sleep at night hungry because there was not enough food?

Katika wiki nne zilizopita, je! Wewe au mtu yeyote wa kaya alilala usiku akiwa na njaa kwa sababu hakukuwa na chakula cha kutosha?

8a. Yes/ Ndio

8b. No/ Hapana

8b. If yes, how often did this happen?

Kama ndio, Hii ilitokea mara ngapi?

8b.1. Rarely (One or twice in the past four weeks)/ Nadra (mara moja au mara mbili kwa wiki 4)

8b.2. Sometimes (three to ten times in the past four weeks)/ Mara kadhaa (mara 3-10 ndani ya wiki 4)

8b.3. Often (more than ten times in the past four weeks)/ Mara nyingi (Zaidi ya mara 10 ndani ya wiki 4)

1. In the past four weeks, did you or any household member go a whole day and night without eating anything because there was not enough food?

Katika wiki nne zilizopita, je! Wewe au mtu yeyote wa kaya alishinda siku nzima na usiku bila kula chochote kwa sababu hakukuwa na chakula cha kutosha?

9a. Yes/ Ndio

9b. No/ Hapana

9b. If yes, how often did this happen?

Kama ndio, Hii ilitokea mara ngapi?

9b.1. Rarely (One or twice in the past four weeks)/ Nadra (mara moja au mara mbili kwa wiki 4)

9b.2. Sometimes (three to ten times in the past four weeks)/ Mara kadhaa (mara 3-10 ndani ya wiki 4)

9b.3. Often (more than ten times in the past four weeks)/ Mara nyingi (Zaidi ya mara 10 ndani ya wiki 4)

**Emergency Care**

*Scenario 1*

Your older mother wakes up and can not move her right arm or leg. Her face is drooping and her speech is slurred.

Mama yako ambaye ni mzee anaamka na hawezi kusogeza mkono au mguu wake wa kulia. Uso wake umeshuka na anachanganya maneno

1. How severe do you think it is?

Unafikiri hii ni mbaya kiasi gani?

1a. Not at all severe/ Sio mbaya kabisa

1b. Possibly severe/ Inawezekana ni mbaya

1c. Somewhat severe/ Ni mbaya kiasi

1d. Severe/ Ni mbaya

1e. Very Severe/ Ni mbaya sana

2. Would you sek care for this?

Je, ungetafuta huduma kwa ajili yah ii?

2a. Yes/ Ndio

2b. No/ Hapana

3. If yes, where would you seek care?

Kama ni ndiyo, ungetafuta wapi huduma?

3a. Dispensary/ Zahanati

3b. Health center/ Kutuo cha afya

3c. Clinic/ Kliniki

3d. Hospital/ Hospitali

3e. Name/ Jina

3f. Traditional healer/ Mganga wa kienyeji

3g. Faith healer/Mponyaji wa kiimani

3h. Pharmacy/ Duka la dawa

3i. Small shop or over-the-counter medications/ Dawa katika duka la kawaida

4. How quickly would you seek care?

Ungetafuta huduma kwa haraka kiasi gani?

4a. Immediately/ Mara moja/bila kukawia

4b. Less than a day/ Chini ya siku moja

4c. Between 1-2 days/ Kati ya siku 1-2

4d. 3 days or more/ Siku 3 au zaidi

*Scenario 2*

Your 14-year-old son has very bad pain in his stomach and can not stop vomiting and crying. He has not eaten in 3 days and refuses water for 2 days.

Mtoto wako wa kiume mwenye miaka 14 ana maumivu makali ya tumbo na anatapika na kulia kwa muda mrefu bila kuacha. Hajala chakula kwa siku 3 na amekataa maji kwa siku 2.

1. How severe do you think it is?

Unafikiri hii ni mbaya kiasi gani?

1a. Not at all severe/ Sio mbaya kabisa

1b. Possibly severe/ Inawezekana ni mbaya

1c. Somewhat severe/ Ni mbaya kiasi

1d. Severe/ Ni mbaya

1e. Very Severe/ Ni mbaya sana

2. Would you sek care for this?

Je, ungetafuta huduma kwa ajili yah ii?

2a. Yes/ Ndio

2b. No/ Hapana

3. If yes, where would you seek care?

Kama ni ndiyo, ungetafuta wapi huduma?

3a. Dispensary/ Zahanati

3b. Health center/ Kutuo cha afya

3c. Clinic/ Kliniki

3d. Hospital/ Hospitali

3e. Name/ Jina

3f. Traditional healer/ Mganga wa kienyeji

3g. Faith healer/Mponyaji wa kiimani

3h. Pharmacy/ Duka la dawa

3i. Small shop or over-the-counter medications/ Dawa katika duka la kawaida

4. How quickly would you seek care?

Ungetafuta huduma kwa haraka kiasi gani?

4a. Immediately/ Mara moja/bila kukawia

4b. Less than a day/ Chini ya siku moja

4c. Between 1-2 days/ Kati ya siku 1-2

4d. 3 days or more/ Siku 3 au zaidi

*Scenario 3*

Your 4-month-old has diarrhea, vomiting and very high fever for 2 days. She is sleeping all day, not drinking milk, and now you can not wake her up.

Mtoto wako wa kike wa miezi 4 anaharisha, kutapika na ana homa kali kwa siku 2. Analala siku nzima, hanywi maziwa na sasa hivi huwezi kumuamsha.

1. How severe do you think it is?

Unafikiri hii ni mbaya kiasi gani?

1a. Not at all severe/ Sio mbaya kabisa

1b. Possibly severe/ Inawezekana ni mbaya

1c. Somewhat severe/ Ni mbaya kiasi

1d. Severe/ Ni mbaya

1e. Very Severe/ Ni mbaya sana

2. Would you sek care for this?

Je, ungetafuta huduma kwa ajili yah ii?

2a. Yes/ Ndio

2b. No/ Hapana

3. If yes, where would you seek care?

Kama ni ndiyo, ungetafuta wapi huduma?

3a. Dispensary/ Zahanati

3b. Health center/ Kutuo cha afya

3c. Clinic/ Kliniki

3d. Hospital/ Hospitali

3e. Name/ Jina

3f. Traditional healer/ Mganga wa kienyeji

3g. Faith healer/Mponyaji wa kiimani

3h. Pharmacy/ Duka la dawa

3i. Small shop or over-the-counter medications/ Dawa katika duka la kawaida

4. How quickly would you seek care?

Ungetafuta huduma kwa haraka kiasi gani?

4a. Immediately/ Mara moja/bila kukawia

4b. Less than a day/ Chini ya siku moja

4c. Between 1-2 days/ Kati ya siku 1-2

4d. 3 days or more/ Siku 3 au zaidi

*Scenario 4*

Your father wakes up with very bad chest pain and trouble breathing.

Baba yako anaamka na maumivu sana ya kifua na anahema kwa shida.

1. How severe do you think it is?

Unafikiri hii ni mbaya kiasi gani?

1a. Not at all severe/ Sio mbaya kabisa

1b. Possibly severe/ Inawezekana ni mbaya

1c. Somewhat severe/ Ni mbaya kiasi

1d. Severe/ Ni mbaya

1e. Very Severe/ Ni mbaya sana

2. Would you sek care for this?

Je, ungetafuta huduma kwa ajili yah ii?

2a. Yes/ Ndio

2b. No/ Hapana

3. If yes, where would you seek care?

Kama ni ndiyo, ungetafuta wapi huduma?

3a. Dispensary/ Zahanati

3b. Health center/ Kutuo cha afya

3c. Clinic/ Kliniki

3d. Hospital/ Hospitali

3e. Name/ Jina

3f. Traditional healer/ Mganga wa kienyeji

3g. Faith healer/Mponyaji wa kiimani

3h. Pharmacy/ Duka la dawa

3i. Small shop or over-the-counter medications/ Dawa katika duka la kawaida

4. How quickly would you seek care?

Ungetafuta huduma kwa haraka kiasi gani?

4a. Immediately/ Mara moja/bila kukawia

4b. Less than a day/ Chini ya siku moja

4c. Between 1-2 days/ Kati ya siku 1-2

4d. 3 days or more/ Siku 3 au zaidi

*Scenario 5*

You are in a bad motorcycle crash and hit your head. Your left arm is broken and you can not breath well.

Uko kwenye ajali mbaya ya pikipiki na umegonga kichwa chako. Mkono wako wa kushoto umevunjika na hauwezi kuhema vizuri.

1. How severe do you think it is?

Unafikiri hii ni mbaya kiasi gani?

1a. Not at all severe/ Sio mbaya kabisa

1b. Possibly severe/ Inawezekana ni mbaya

1c. Somewhat severe/ Ni mbaya kiasi

1d. Severe/ Ni mbaya

1e. Very Severe/ Ni mbaya sana

2. Would you sek care for this?

Je, ungetafuta huduma kwa ajili yah ii?

2a. Yes/ Ndio

2b. No/ Hapana

3. If yes, where would you seek care?

Kama ni ndiyo, ungetafuta wapi huduma?

3a. Dispensary/ Zahanati

3b. Health center/ Kutuo cha afya

3c. Clinic/ Kliniki

3d. Hospital/ Hospitali

3e. Name/ Jina

3f. Traditional healer/ Mganga wa kienyeji

3g. Faith healer/Mponyaji wa kiimani

3h. Pharmacy/ Duka la dawa

3i. Small shop or over-the-counter medications/ Dawa katika duka la kawaida

4. How quickly would you seek care?

Ungetafuta huduma kwa haraka kiasi gani?

4a. Immediately/ Mara moja/bila kukawia

4b. Less than a day/ Chini ya siku moja

4c. Between 1-2 days/ Kati ya siku 1-2

4d. 3 days or more/ Siku 3 au zaidi

**Health Utilization and barriers**

1. Did you or anyone else in your household have a health emergency in the last year?

Je, wewe au mwinginie yeyote kwenye kaya yenu alipata dharua ya kiafya katika mwaka uliopita?

1a. If yes:

Kma ndio:

1a.1 Did any of these people die?/ Je, kuna yoyote kati ya hawa aliyekufa?

1a.2 If yes, where did they die? / Kama ndio, wamekufa

1. Emergency 1 (need to specify if death or not)

Dharua 1

2a. Which individual in the household? / Ni nani katika kaya?

2b. Approximately when did this happen? / Kwa kukadiria ilitokea lini?

2c. What type of symptoms did you or they have? / Ulikua au walikuwa na dalili zipi?

2c.1 Motor vehicle accident/ aiali va gari

2c.2 Chest pain/ maumivu va kifua

2c.3 Difficulty breathing/ kupumua kwa shida

2c.4 Sudden unilateral paralysis/ kupooza ghafla upande mmoja

2c.5 Trouble speaking/ kuzungumza kwa shida

2c.6 Confusion/ kuchanganyikiwa

2c.7 Vision problems/ matattizo ya kuona

2c.8 Severe pain/ maumivu makali

2c.9 Snake or animal or insect bite / nyoka / mnyama / kuumwa na mdudu

2c.10 Penetrating trauma/ jeraha lililochimbika

2c.11 Burn/ moto/kuungua

2c.12 Fever/ homa

2c.13 Nausea/vomiting/diarrhea/ kichefuchefu/kutapika/kuharisha

2c.14 Other/ nyingine

2d. Did you seek healthcare for this condition? / Je, ulitafuta huduma ya afya kwa ajili ya hali hii?

2d.1 Yes/ Ndio

2d.2 No/ Hapana

2e. If yes, how long were they having symptoms before seeking care? / Kama ndio, Amekuwa na dalili kwa muda gani kabla ya kutafuta huduma ya afya?

2e.1 Hours / Masaa

2e.2 Where did you go initially? / Ulienda wapi mara ya kwanza?

2e.2.a Dispensary / Zahanati

2e.2.b Health center/ Kituo cha afya

2e.2.c Clinic/ Kliniki

2e.2.d Hospital/ Hospitali

2e.2.e Name/ Jina

2e.2.f Traditional healer/ Mganga wa kienyeji

2e.2.g Faith healer/Mponyaji wa kiimani

2e.2.h Pharmacy/ Duka la dawa

2e.2.i Small shop or over-the-counter medications/ Dawa katika duka la kawaida

2e.3 Why did you choose to go there? / Ni kituo cha karibu

2e.3.a Closest facility / ni kituo cha karibu

2e.3.b Familiarity / mazoea

2e.3.c Cost / gharama

2e.3.d Quality of care/ ubora wa huduma

2e.3.e Care from a specific doctor / huduma kutoka kwa daktarin Fulani

2e.3.f Covered by insurance / kuna bima

2e.3.g Available specialty / upatikanaji wa utaalamu

2e.4 How did you get there? / Ulifikaje hapo?

2e.4.a Car (private) / Gari (binafsi)

2e.4.b Car (taxi) / Gari (taxi)

2e.4.c Bajaji / Bajaji

2e.4.d Motorcycle / Pikipiki

2e.4.e Dala Dala / Daladala

2e.4.f Bicycle / Baiskeli

2e.4.g Large bus / Basi kubwa

2e.4.h Walked / Kutembea

2e.5 How long did it take you to get there? / Ilikuchukua muda gani kufika hapo?

2e.5.a Hours / Msaa

2e.5.b Days / Siku

2e.6 Were you transferred to another facility? / Je, ulihamishiwa katika kituo kingine?

2e.6.a Yes / Ndio

2e.6.b No/ Hapana

2e.7 If yes, where? / Kama ndiyo, wapi?

2e.8 How many times were you transferred before your final destination? / Je, umehamishwa mara ngapi kabla hujafika kituo cha mwisho?

2e.9 How did you get there? / Ulifikaje hapo?

2e.9.a Car (private) / Gari (binafsi)

2e.9.b Car (taxi) / Gari (taxi)

2e.9.c Bajaji / Bajaji

2e.9.d Motorcycle / Pikipiki

2e.9.e Dala Dala / Daladala

2e.9.f Bicycle / Baiskeli

2e.9.g Large bus / Basi kubwa

2e.9.h Walked / Kutembea

2e.10 How long from when you decided to seek care to when you reached the final facility? / Ilikuchukua muda gani tangu kuamua kutafuta huduma mpaka kufika katika kituo cha mwisho?

2e.10.a Hours / Msaa

2e.10.b Days / Siku

2e.11 How long from the time you arrived until you were seen by a doctor or nurse at the final facility? / Ilikuchukua muda gani tangu kufika mpaka umuone daktarin au nesi katika kituo cha mwisho?

2e.11.a Hours / Msaa

2e.11.b Days / Siku

1. Emergency 2 (need to specify if death or not)

Dharua 2

3a. Which individual in the household? / Ni nani katika kaya?

3b. Approximately when did this happen? / Kwa kukadiria ilitokea lini?

3c. What type of symptoms did you or they have? / Ulikua au walikuwa na dalili zipi?

3c.1 Motor vehicle accident/ aiali va gari

3c.2 Chest pain/ maumivu va kifua

3c.3 Difficulty breathing/ kupumua kwa shida

3c.4 Sudden unilateral paralysis/ kupooza ghafla upande mmoja

3c.5 Trouble speaking/ kuzungumza kwa shida

3c.6 Confusion/ kuchanganyikiwa

3c.7 Vision problems/ matattizo ya kuona

3c.8 Severe pain/ maumivu makali

3c.9 Snake or animal or insect bite / nyoka / mnyama / kuumwa na mdudu

3c.10 Penetrating trauma/ jeraha lililochimbika

3c.11 Burn/ moto/kuungua

3c.12 Fever/ homa

3c.13 Nausea/vomiting/diarrhea/ kichefuchefu/kutapika/kuharisha

3c.14 Other/ nyingine

3d. Did you seek healthcare for this condition? / Je, ulitafuta huduma ya afya kwa ajili ya hali hii?

3d.1 Yes/ Ndio

3d.2 No/ Hapana

3e. If yes, how long were they having symptoms before seeking care? / Kama ndio, Amekuwa na dalili kwa muda gani kabla ya kutafuta huduma ya afya?

3e.1 Hours / Masaa

3e.2 Where did you go initially? / Ulienda wapi mara ya kwanza?

3e.2.a Dispensary / Zahanati

3e.2.b Health center/ Kituo cha afya

3e.2.c Clinic/ Kliniki

3e.2.d Hospital/ Hospitali

3e.2.e Name/ Jina

3e.2.f Traditional healer/ Mganga wa kienyeji

3e.2.g Faith healer/Mponyaji wa kiimani

3e.2.h Pharmacy/ Duka la dawa

3e.2.i Small shop or over-the-counter medications/ Dawa katika duka la kawaida

3e.3 Why did you choose to go there? / Ni kituo cha karibu

3e.3.a Closest facility / ni kituo cha karibu

3e.3.b Familiarity / mazoea

3e.3.c Cost / gharama

3e.3.d Quality of care/ ubora wa huduma

3e.3.e Care from a specific doctor / huduma kutoka kwa daktarin Fulani

3e.3.f Covered by insurance / kuna bima

3e.3.g Available specialty / upatikanaji wa utaalamu

3e.4 How did you get there? / Ulifikaje hapo?

3e.4.a Car (private) / Gari (binafsi)

3e.4.b Car (taxi) / Gari (taxi)

3e.4.c Bajaji / Bajaji

3e.4.d Motorcycle / Pikipiki

3e.4.e Dala Dala / Daladala

3e.4.f Bicycle / Baiskeli

3e.4.g Large bus / Basi kubwa

3e.4.h Walked / Kutembea

3e.5 How long did it take you to get there? / Ilikuchukua muda gani kufika hapo?

3e.5.a Hours / Msaa

3e.5.b Days / Siku

3e.6 Were you transferred to another facility? / Je, ulihamishiwa katika kituo kingine?

3e.6.a Yes / Ndio

3e.6.b No/ Hapana

3e.7 If yes, where? / Kama ndiyo, wapi?

3e.8 How many times were you transferred before your final destination? / Je, umehamishwa mara ngapi kabla hujafika kituo cha mwisho?

3e.9 How did you get there? / Ulifikaje hapo?

3e.9.a Car (private) / Gari (binafsi)

3e.9.b Car (taxi) / Gari (taxi)

3e.9.c Bajaji / Bajaji

3e.9.d Motorcycle / Pikipiki

3e.9.e Dala Dala / Daladala

3e.9.f Bicycle / Baiskeli

3e.9.g Large bus / Basi kubwa

3e.9.h Walked / Kutembea

3e.10 How long from when you decided to seek care to when you reached the final facility? / Ilikuchukua muda gani tangu kuamua kutafuta huduma mpaka kufika katika kituo cha mwisho?

3e.10.a Hours / Msaa

3e.10.b Days / Siku

3e.11 How long from the time you arrived until you were seen by a doctor or nurse at the final facility? / Ilikuchukua muda gani tangu kufika mpaka umuone daktarin au nesi katika kituo cha mwisho?

3e.11.a Hours / Msaa

3e.11.b Days / Siku

1. Emergency 3 (need to specify if death or not)

Dharua 3

4a. Which individual in the household? / Ni nani katika kaya?

4b. Approximately when did this happen? / Kwa kukadiria ilitokea lini?

4c. What type of symptoms did you or they have? / Ulikua au walikuwa na dalili zipi?

4c.1 Motor vehicle accident/ aiali va gari

4c.2 Chest pain/ maumivu va kifua

4c.3 Difficulty breathing/ kupumua kwa shida

4c.4 Sudden unilateral paralysis/ kupooza ghafla upande mmoja

4c.5 Trouble speaking/ kuzungumza kwa shida

4c.6 Confusion/ kuchanganyikiwa

4c.7 Vision problems/ matattizo ya kuona

4c.8 Severe pain/ maumivu makali

4c.9 Snake or animal or insect bite / nyoka / mnyama / kuumwa na mdudu

4c.10 Penetrating trauma/ jeraha lililochimbika

4c.11 Burn/ moto/kuungua

4c.12 Fever/ homa

4c.13 Nausea/vomiting/diarrhea/ kichefuchefu/kutapika/kuharisha

4c.14 Other/ nyingine

4d. Did you seek healthcare for this condition? / Je, ulitafuta huduma ya afya kwa ajili ya hali hii?

4d.1 Yes/ Ndio

4d.2 No/ Hapana

4e. If yes, how long were they having symptoms before seeking care? / Kama ndio, Amekuwa na dalili kwa muda gani kabla ya kutafuta huduma ya afya?

4e.1 Hours / Masaa

4e.2 Where did you go initially? / Ulienda wapi mara ya kwanza?

4e.2.a Dispensary / Zahanati

4e.2.b Health center/ Kituo cha afya

4e.2.c Clinic/ Kliniki

4e.2.d Hospital/ Hospitali

4e.2.e Name/ Jina

4e.2.f Traditional healer/ Mganga wa kienyeji

4e.2.g Faith healer/Mponyaji wa kiimani

4e.2.h Pharmacy/ Duka la dawa

4e.2.i Small shop or over-the-counter medications/ Dawa katika duka la kawaida

4e.3 Why did you choose to go there? / Ni kituo cha karibu

4e.3.a Closest facility / ni kituo cha karibu

4e.3.b Familiarity / mazoea

4e.3.c Cost / gharama

4e.3.d Quality of care/ ubora wa huduma

4e.3.e Care from a specific doctor / huduma kutoka kwa daktarin Fulani

4e.3.f Covered by insurance / kuna bima

4e.3.g Available specialty / upatikanaji wa utaalamu

4e.4 How did you get there? / Ulifikaje hapo?

4e.4.a Car (private) / Gari (binafsi)

4e.4.b Car (taxi) / Gari (taxi)

4e.4.c Bajaji / Bajaji

4e.4.d Motorcycle / Pikipiki

4e.4.e Dala Dala / Daladala

4e.4.f Bicycle / Baiskeli

4e.4.g Large bus / Basi kubwa

4e.4.h Walked / Kutembea

4e.5 How long did it take you to get there? / Ilikuchukua muda gani kufika hapo?

4e.5.a Hours / Msaa

4e.5.b Days / Siku

4e.6 Were you transferred to another facility? / Je, ulihamishiwa katika kituo kingine?

4e.6.a Yes / Ndio

4e.6.b No/ Hapana

4e.7 If yes, where? / Kama ndiyo, wapi?

4e.8 How many times were you transferred before your final destination? / Je, umehamishwa mara ngapi kabla hujafika kituo cha mwisho?

4e.9 How did you get there? / Ulifikaje hapo?

4e.9.a Car (private) / Gari (binafsi)

4e.9.b Car (taxi) / Gari (taxi)

4e.9.c Bajaji / Bajaji

4e.9.d Motorcycle / Pikipiki

4e.9.e Dala Dala / Daladala

4e.9.f Bicycle / Baiskeli

4e.9.g Large bus / Basi kubwa

4e.9.h Walked / Kutembea

4e.10 How long from when you decided to seek care to when you reached the final facility? / Ilikuchukua muda gani tangu kuamua kutafuta huduma mpaka kufika katika kituo cha mwisho?

4e.10.a Hours / Msaa

4e.10.b Days / Siku

4e.11 How long from the time you arrived until you were seen by a doctor or nurse at the final facility? / Ilikuchukua muda gani tangu kufika mpaka umuone daktarin au nesi katika kituo cha mwisho?

4e.11.a Hours / Msaa

4e.11.b Days / Siku

**Healthcare needs**

1. If you or a family member has another health emergency, what is the number one thing that would make it easier to go to the hospital?

Je, kama wewe au mwanafamilia wako dharuranyingine ya afya, je ni kitu gani cha kwanza ambacho kitafanya kufika hospitali iwe rahisi?

1a. Provision of transportation / Kupata usafiri

1b. Lower costs of healthcare / Kupunguza gharama za huduma ya afya

1c. Family or friend available to help / Kuwa na familia au Rafiki wa kukusaidia

1d. Better understanding of hospital capabilities / Uelewa mzuri Zaidi wa uwezo wa hospitali

1e. Closer facility in my community / Kuwa na kituo cha afya au hospitali karibu Zaidi na nyumbani

1f. Better roads / Barabara nzuri

1g. Improved hours of local facility / Uboreshaji wa masaa katika vituo vya karibu

1. Do you have a cell phone or access to a cell phone for most of the day?

Je, una simu au unaweza kupata simu ya kutumia kwa karibu siku nzima?

2a. Yes / Ndio

2b. No / Hapana

1. Are you always able to use your cell phone at all times?

Una uweo wa kutumia simu yako wakati wote?

3a. Yes / Ndio

3b. No / Hapana

3b.1 If no, why not? / kama hapana, kwa nini?

3b.1.a Poor coverage / Hakuna mtandao kwetu

3b.1.b Battery charge / Chaji ya betri

3b.1.c Lack of air/talk time / kukosa vocha/dakika za mongezi

**Snakebites**

1. Have you ever suffered a snakebite?

Je, umewahi kung’atwa na nyoka?

1a. Yes / Ndio

1b. No / Hapana

1. Did you seek care?

Je, ulitafuta huduma?

2a. Yes / Ndio

2b. No / Hapana

1. If yes, where?

Kama ndio, wapi?

3a. Dispensary / Zahanati

3b. Health center/ Kituo cha afya

3c. Clinic/ Kliniki

3d. Hospital/ Hospitali

3e. Name/ Jina

3f. Traditional healer/ Mganga wa kienyeji

3g. Faith healer/Mponyaji wa kiimani

3h. Pharmacy/ Duka la dawa

3i. Small shop or over-the-counter medications/ Dawa katika duka la kawaida

1. Did you take anything to treat this at home, from a traditional healer or any other member of the health system?

Je, ulitumia kitu chochote kutibu hili ukiwa nyumbani, kutoka kwa mganga wa kienyeji au yoyote mwingine kwenye mfumo wa afya?

4a. Yes / Ndio

4b. No / Hapana

1. If yes,

Kama ndio,

5a. Was it something from home? / kilikuwa ni kitu kutoka nyumbani?

5a.1 Yes / Ndio

5a.2 No / Hapana

5b. Was it something from a traditional healer? / Kilikuwa ni kitu kutoka kwa mganga wa kienyeji?

5b.1 Yes / Ndio

5b.2 No / Hapana

5c. Was it medication? / Je, ilikuwa ni dawa kutoka kwa daktarin au famasia?

5c.1 Yes / Ndio

5c.2 No / Hapana

1. If Yes, was it from a:

Kama ndio, ilitoka kwa:

6a. Health center/ Kituo cha afya

6b. Pharmacy/ Duka la dawa

6c. Dispensary/ Zahanati

1. Did you receive antivenom?

Je, ulipewa kikinga sumu?

7a. Yes / Ndio

7b. No / Hapana

1. Did you fully recover to your prior health?

Je, ulirejea kikamilifu katika hali yako ya afya ya kabla?

8a. Yes / Ndio

8b. No / Hapana

1. If no, does the snake bite limit your ability to work (including at home)?

Kama hapana, kung’atwa na nyoka kumezuia uwezekano wako wa kufanya kazi (hata kazi za nyumbani)?

9a. Yes / Ndio

9b. No / Hapana

1. If yes, how long did it take you to fully recover?

Kama ndio, ilikuchukua muda gani mpaka kupona kabisa?

10a.Number of days or number of months or number of years / Siku ngapi au Miezi mingapi au Miaka mingapi

1. List three things you cannot do after your snakebite

Taja vitu vitatu ambavyo huwezi kuvifanya baada ya kung’atwa na nyoka

1. Has anyone in your household ever suffered a snakebite?

Je, kuna mtu yoyote katika kaya yako ambaye amewahi kung’atwa na nyonka?

12a. Yes / Ndio

12b. No / Hapana

1. Did they seek care?

Alitafuta huduma?

13a. Yes / Ndio

13b. No / Hapana

1. If yes, where?

Kama ndio, wapi?

14a. Dispensary / Zahanati

14b. Health center/ Kituo cha afya

14c. Clinic/ Kliniki

14d. Hospital/ Hospitali

14e. Name/ Jina

14f. Traditional healer/ Mganga wa kienyeji

14g. Faith healer/Mponyaji wa kiimani

14h. Pharmacy/ Duka la dawa

14i. Small shop or over-the-counter medications/ Dawa katika duka la kawaida

1. Did they take anything to treat this at home, from a traditional healer or any other member of the health system?

Je, alitumia kitu chochote kutibu hili ukiwa nyumbani, kutoka kwa mganga wa kienyeji au yoyote mwingine kwenye mfumo wa afya?

15a. Yes / Ndio

15b. No / Hapana

1. If yes,

Kama ndio,

16a. Was it something from home? / kilikuwa ni kitu kutoka nyumbani?

16a.1 Yes / Ndio

16a.2 No / Hapana

16b. Was it something from a traditional healer? / Kilikuwa ni kitu kutoka kwa mganga wa kienyeji?

16b.1 Yes / Ndio

16b.2 No / Hapana

16c. Was it medication? / Je, ilikuwa ni dawa kutoka kwa daktarin au famasia?

16c.1 Yes / Ndio

16c.2 No / Hapana

1. If Yes, was it from a:

Kama ndio, ilitoka kwa:

17a. Health center/ Kituo cha afya

17b. Pharmacy/ Duka la dawa

17c. Dispensary/ Zahanati

1. Did they receive antivenom?

Je, alipewa kikinga sumu?

18a. Yes / Ndio

18b. No / Hapana

1. If no,

Kama hapana,

1. Did they take a prescribed medicine?

Je, alitumia dawa yoyote ya kuandikiwa?

20a. Yes / Ndio

20b. No / Hapana

1. Did they take anything from a traditional healer?

Je, alitumia kitu chochote utoka kwa mganga wa kienyeji?

21a. Yes / Ndio

21b. No / Hapana

1. Did they take anything at home?

Je, alitumia kitu chochote akiwa nyumbani?

22a. Yes / Ndio

22b. No / Hapana

1. Did they fully recover to their prior health?

Je, alipona kabisa na kurudi kwenye afya yao ya kabla?

23a. Yes / Ndio

23b. No / Hapana

1. If no, does the snake bite limit their ability to work (including at home)?

Kama hapana, kung’atwa na nyoka kunamzuia kufanya kazi (hata akiwa nyumbani)?

24a. Yes / Ndio

24b. No / Hapana

1. If yes, how long did it take them to fully recover?

Kama ndio, ilimchukua muda gani mpaka kupona kabisa?

25a.Number of days or number of months or number of years / Siku ngapi au Miezi mingapi au Miaka mingapi

**AUDIT**

1. How often during the last year do you have a drink containing alcohol?

Kwa mwaka uliopita ni mara ngapi unatumia kinywaji kilicho na kilevi?

1a. Never / Hakuna

1b. Monthly or less / Kila mwezi au chini ya mwezi

1c. 2-4 times a month / Mara 2 hadi 4 mwezi

1d. 2-3 times a week / Mara 2 hadi 3 wiki

1e. 4 or more times week / 4 au Zaidi wiki

1. How many drinks containing alcohol do you have on a typical day when you are drinking during the last year?

Kwa mwaka uliopita kwa siku ya kawaidia unatumia vinywaji vingapi vyenye kilevi unapokuwa unakunywa?

2a. 1 - 2 / 1 au 2

2b. 3 - 4 / 3 au 4

2c. 5 - 6 / 5 au 6

2d. 7 - 9 / 7 au 9

2e. 10 or more / 10 au zaidi

1. Do you have six or more drinks on one ocassion?

Kwa mwaka uliopita ni mara ngapi unatumia vinywaji sita au Zaidi kwa mara moja kwa mwaka uliopita?

3a. Never / Hakuna

3b. Less than monthly / Chini ya kila mwezi

3c. Monthly / kila mwezi

3d. Weekly / Kwa wiki

3e. Daily almost dialy / kila siku au karibu kila siku

1. How often during the last year have you found that you were not able to stop drinking once you had started?

Mara ngapi katika mwaka uliopita uligundua hukuweza kuacha kunywa vinywaji vyenye kilevi ukishaanza kutumia?

4a. Never / Hakuna

4b. Less than monthly / Chini ya kila mwezi

4c. Monthly / kila mwezi

4d. Weekly / Kwa wiki

4e. Daily almost dialy / kila siku au karibu kila siku

1. How often during the last year have you failed to do what was normally expected of you because of drinking?

Mara ngapi katika mwaka uliopita ulishindwa kufanya unavyotarajiwa kutoka kwako kwa sabadu ya kunywa vinywaji vyenye kilevi?

5a. Never / Hakuna

5b. Less than monthly / Chini ya kila mwezi

5c. Monthly / kila mwezi

5d. Weekly / Kwa wiki

5e. Daily almost dialy / kila siku au karibu kila siku

1. How often during the last year have you needed a first drinkin the morning to get yourself going after a heavy drinking session?

Mara ngapi katika mwaka uliopita ulihitaji kinywaji cha kwanza asubuhi ili kuweza kuendelea na shughuli zako baada ya kunywa sana?

6a. Never / Hakuna

6b. Less than monthly / Chini ya kila mwezi

6c. Monthly / kila mwezi

6d. Weekly / Kwa wiki

6e. Daily almost dialy / kila siku au karibu kila siku

1. How often during the last year have you had a feeling of guilt or remorse after drinking?

Mara ngapi katika mwaka uliopita ulijihisi kuwa na hatia au kujilaumu baada ya kunywa?

7a. Never / Hakuna

7b. Less than monthly / Chini ya kila mwezi

7c. Monthly / kila mwezi

7d. Weekly / Kwa wiki

7e. Daily almost dialy / kila siku au karibu kila siku

1. How often during the last year have you been unable to remember what happened the night before because of your drinking?

Mara ngapi katika mwaka uliopita hukuweza kukumbuka kilichotendeka usiku uliopita kwa sababu ulikunywa?

8a. Never / Hakuna

8b. Less than monthly / Chini ya kila mwezi

8c. Monthly / kila mwezi

8d. Weekly / Kwa wiki

8e. Daily almost dialy / kila siku au karibu kila siku

1. Have you or someone else been injured because of your drinking?

Je, umejeruhiwa au mtu mwingine kujeruhiwa kwa sababu ya kunywa kwako?

9a. No / Hapana

9b. Yes, but not in the last year / Ndiyo, lakini si kwa mwaka

9c. Yes, during the last year / Ndiyo kwa mwaka uliopita

1. Has a relative, friend, doctor, or other health care worker been concerned about your drinking or suggested you to cut down?

Je, ndugu yako au Rafiki yako au daktarin au mhudumu wa afya mwingine ameguswa na kunywa kwako au kupendekeza upunguze kunywa kwako?

10a. No / Hapana

10b. Yes, but not in the last year / Ndiyo, lakini si kwa mwaka

10c. Yes, during the last year / Ndiyo kwa mwaka uliopita
