# Supplementary material for: Emergency care knowledge, utilization, and barriers in Northern Tanzania: A community-based survey
Source: PLOS Glob Public Health. 2025 Jan 6;5(1):e0004032. doi: 10.1371/journal.pgph.0004032 (PMC11703045; doi:10.1371/journal.pgph.0004032)
Supplement: Attachment — Submitted filename: Response to reviewers.docx [file pgph.0004032.s004.docx]

1. Please include a complete copy of PLOS’ questionnaire on inclusivity in global research in your revised manuscript. Our policy for research in this area aims to improve transparency in the reporting of research performed outside of researchers’ own country or community. The policy applies to researchers who have travelled to a different country to conduct research, research with Indigenous populations or their lands, and research on cultural artefacts. The questionnaire can also be requested at the journal’s discretion for any other submissions, even if these conditions are not met. Please find more information on the policy and a link to download a blank copy of the questionnaire here: [https://journals.plos.org/globalpublichealth/s/best-practices-in-research-reporting](https://nam11.safelinks.protection.outlook.com/?url=https%3A%2F%2Fjournals.plos.org%2Fglobalpublichealth%2Fs%2Fbest-practices-in-research-reporting&data=05%7C02%7Canjni.joiner%40duke.edu%7C77351615abf048f5143908dcc6ff87f1%7Ccb72c54e4a314d9eb14a1ea36dfac94c%7C0%7C0%7C638604048420914298%7CUnknown%7CTWFpbGZsb3d8eyJWIjoiMC4wLjAwMDAiLCJQIjoiV2luMzIiLCJBTiI6Ik1haWwiLCJXVCI6Mn0%3D%7C0%7C%7C%7C&sdata=XsuvJBpBsQ37wzOIbh00joIOtS91CFSAc6oZEO3grFw%3D&reserved=0). Please upload a completed version of your questionnaire as Supporting Information when you resubmit your manuscript.

*Thank you, we have completed and uploaded this questionnaire.*

*All line number references below refer to the “Manuscript_tracked changes” document.*

**Reviewer #1**: Thank you for allowing me to review this abstract. Overall, this is a very important household survey assessing health literacy and access to emergency care.

*Thank you for your kind words and attention to this manuscript.*

Abstract: background or results, consider adding additional descriptive data about the community that was interviewed as well as the health system eg income brackets, rural vs urban, number of facilities and/or distance to facility

*Thank you for this suggestion please see line 45 and 46, we have now included insurance coverage and household income, as it reflects the gap in health financing that has been discussed in the discussion section. Language added includes: “73.7% used cash and/or had no insurance. The mean monthly household income was 226,107.6 Tanzanian Shillings.”*

Methods:

line 100 “The three districts share a total of 13 hospitals” Please provide more details about the types and distribution of facilities, is there any data on proximity to facilities for the community. What types of services are available at each level/type of facility? Many readers will not understand tiered systems and these can vary significantly by region in terms of resources.

*Thank you for highlighting this, we have added services provided in these facilities (lines 119-122) however, during the data collection process, distance from health facilities to households was not kept in consideration.*

Line 125: how were households randomly sampled?

*Additional information regarding household sampling was added with the following language: “A random starting point was generated in each village using QGIS 2.18.7. A 1 km^2^ polygon was placed around this starting point and 20 random points were generated within this polygon.” (lines 144-146)*

The headers in methods are a bit confusing, sample size and sampling could be split into sample size and then a new section combining the sampling techniques, labeled as such (rather than study protocols) eg Selection of households

*Thank you for this suggestion. We have restructured the manuscript to include the following headings: Sample Size and Sampling Techniques.*

Line 171: Why snakebites, I would assume the survey was multi-purpose, briefly explain this

*Thanks for raising this. Yes, the survey was multipurpose and one of our team members was interested in collecting some preliminary data on the burden of snakebites in this region. We added clarification with the following languages (line 193): “Given the multi-purpose use of the survey, additional questions were asked regarding alcohol use, food insecurity, and snake bites.”*

Line 175: “traumatic situations” potentially unclear as trauma can be injury, emotional etc, suggestion for “injuries”

*We have added clarification to line 198.*

Lines 180-182: Where respondents choosing from a list of options? Or were free text options available

*Participants selected from a list of options. A reference to Supplement 1 was added (line 204) in order to provide clarity and refer the reader to the survey.*

Results Table 2: What is the rationale behind reporting tribe and religion, if this does nto add to the discussion or explanation, would consider deleting

*Thank you for this important suggestion. Although we did not focus on these variables in the discussion, we think this demonstrates the diversity of the population as well as context to some of the findings on utilization of faith healers.*

Number of household members consider presenting either median (IQR) or mean Q1, Q3, you do not need 4 lines representing the same data, remove missing, report when significant

*We appreciate the suggestion and have revised the tables accordingly, as well as language under “Statistical analysis” (lines 225-227).*

Monthly household income, same comment as household members, report one value and associated measure of variance

*Thank you, we have removed missingness from all tables and revised the results for “Monthly household income” to a single value with variance.*

Residence, only need yes or no line, not both

*Thank you, table 2 has been revised.*

Table 3: missing data can be supplied as text or footnotes to table, row is very distracting in the table

*This is an excellent suggestion. We have revised accordingly.*

Line 243: How is delayed access defined?

*This is an important question. This is defined in lines 215-218 under the “Main outcomes” section: “A delay in reaching healthcare services was defined as either a lack of seeking care or a delay in arrival at the final location of care of more than one hour from the time of initially seeking care”.*

Table 4: overall this table is too big, consider adding header rows or dividing into multiple tables

*We removed some unnecessary rows and revised the table to make it smaller.*

consider adding a row above which household member had an emergency with N that can then carry though for the all the following sections

*Great suggestion - added.*

“how long did it take…” present one measure and it’s variance in same row

*Corrected, thank you.*

line 251: confusing sentence, “individuals with cash personal”

*Thank you for noting this. We have revised the language to: “individuals who used personal cash”. (line 321)*

Table 5:

Difficult accessibility of house-how is this defined?

*This is a great question. We added a footnote to Table 5 defining difficult accessibility, “Difficult accessibility of house was defined as a household on a non-paved road or a household that could not be reached by a four-wheel vehicle. (lines 340-341).”*

Why is number of household members included here, one line with measure and variance

*Thank you, this was revised accordingly.*

Income, suggest one measure with variance in one row, add row with USD conversions for global audience

*Thank you, this was revised accordingly.*

Table 6: same comments as previously listed, decrease number of rows with variables containing mean, median, Q1, Q3 and range

*Thank you, this was revised accordingly.*

Line 268-271, confusing sentence, please clarify

*We agree that this sentence would benefit from additional clarity. We have revised to the following: “While not statistically significant at the 0.05 level of significance, those with inappropriate medical responses had a lower proportion of household emergencies in the previous year (10.7% vs 16.6%) when compared to those with appropriate responses to all questions (p = 0.053). (lines 361-363).”*

Discussion: lines 304-321: the points made are repetitive in these 2 paragraphs. what do the authors recommend in response to this association, is there evidence for successful interventions that improve health literacy

*We thank the reviewer for pointing this out. We have removed the repetitive language and have also provided additional language at the end of the second paragraph regarding successful interventions (lines 438-457).*

Lines 335-339 terms emergency care services and prehospital care are used interchangeably, they are not the same. Why is an ambulance the only potential solution? There have been successful community transport initiatives, mostly in the OB literature

*This is an excellent point. We have modified the language and added a relevant reference highlighting the point you made (lines 474-477).*

The final discussion points on expanding emergency care at facilities and expanding prehospital care are intertwined and may be confusing to non-EM providers. Both are important but this is not entirely clear as written

*The sentence was separated to clarify the distinction between emergency care and prehospital care (lines 474-477).*

Limitations

Line 365: this is the first time that potential language barriers are mentioned, what actions were taken to overcome this, how is the language barrier linked to interpretation of an emergency?

*Thank you. Our study only included those who could fluently speak in Kiswahili (lines 118-119). The language surrounding this limitation was clarified (lines 510-511).*

**Reviewer #2**:

The study addresses a critical need for improving emergency systems in low-resource settings and makes a substantial contribution to public health literature in this area.

*We appreciate your review of this manuscript and your important insights.*

However, there are areas that require clarification and further elaboration, particularly in the methods, results, and discussion sections. The manuscript would benefit from clearer explanations of the sampling strategy, more detailed context regarding income levels and their impact on emergency care, and expanded discussion on policy recommendations for improving emergency care systems in similar settings. **Please find attached comments.**

Line 36: The methodology is quite dense. Simplify it slightly for clarity. Perhaps include a mention that this was a cross-sectional study and leave the details for the main text.

*Thank you, we have shortened the methods section accordingly.*

Line 38: Clarify what you mean by "inappropriate response to any of five hypothetical emergency conditions." For readers not familiar with the study's context, it's important to define "inappropriate response" clearly.

*Thank you for this comment. It was difficult for us to define this within the limited space of the abstract. It is clearly defined within the methods section of the manuscript.*

Lines 68-70: Consider adding a recent reference that discusses the relationship between NCDs and emergency care in Sub-Saharan Africa, as this would ground the importance of the study in a broader context.

*Thank you for raising this important point. We added additional relevant literature and restructured the first paragraph of the background for greater context. (lines 66-68)*

Line 118: The sample size estimation could benefit from more elaboration. Why was a 35% emergency condition incidence chosen? Providing more context will help justify the calculations.

*There is limited literature on the incidence of emergency conditions in this region. We used a previously estimated incidence from Cameroon to justify our sample size as this was the only pertinent literature that we could identify at the time of study planning.*

Line 128: The protocol for identifying villages and households changed midway through the study. Please clarify how the two approaches differ and how this might have impacted the data collection and results.

*Thank you, additional information was added to the limitations. (lines 499-502)*

Line 140: Consider clarifying how households were defined and what happened in cases where multiple families lived in a single structure. This could influence the data on household demographics.

*Thank you for such an important comment on demographics. We previously provided a definition of household within the methods section (lines 166-167).*

Lines 175-180: The five hypothetical emergency conditions are described well. However, it might be helpful to include why these specific scenarios were chosen and whether they reflect common emergencies in the region.

*Great suggestion, further clarification was added regarding the scenarios (lines 198-199).*

Lines 221-225: The description of household income needs to be contextualized. Including a brief mention of what constitutes low, middle, and high income in Tanzania would help readers outside the region.

*Thank you, data on average annual income was added to the Methods-Study Design and Setting section (lines 125-126).*

Table 2: Some tables (e.g., Table 2 and 3) could be simplified for readability. Consider consolidating some rows or providing a more straightforward way to present the data...

*We appreciate this suggestion. This was also noted by another reviewer. We have accordingly simplified the tables and removed unnecessary lines.*

Line 239: The high percentage of respondents who would seek care immediately is a positive finding. However, consider discussing why some might still delay care, as this can provide a more balanced understanding of the barriers.

*Thank you for this suggestion. Additional information was added to the discussion to better hypothesize the reasons for these delays using existing literature.*

Line 248: The association between delayed access and insurance status is significant. It would be interesting to expand on why insurance plays such a critical role in this context and how it might differ from other regions in Tanzania or Sub-Saharan Africa.

*Thank you for such an important highlight, this has been discussed however in the discussion section with references from Rwanda and Ghana. We also added an additional reference and line regarding a recent study from Burkina Faso. (lines 468-470).*

Lines 277-283: Expand on how community education could specifically address gaps in emergency care knowledge. Practical examples of what such an education campaign could look like would add value.

*We appreciate this suggestion. Additional information was added to the conclusion to address this (lines 519-521).*

Line 288: The financial burden is a significant factor in care-seeking behavior. It would help to compare Tanzania’s healthcare financing issues with other countries in Sub-Saharan Africa, potentially offering insights into region-wide challenges.

*Thank you for this suggestion. Additional information was added to the discussion to address this. (lines 389-406)*

Lines 323-324: The point about distance and time to care is critical. A more in-depth discussion of how geography affects emergency response, perhaps with a comparison to similar countries, could enhance the discussion.

*We appreciate this highlight. We included a comparison with studies from similar settings cited with reference and added an additional relevant citation. (lines 468-470)*

Lines 355: The limitations are acknowledged but could be strengthened by discussing the potential biases introduced by using different sampling methods across villages. What steps, if any, were taken to ensure comparability?

*Thank you, we added additional information in the limitations.*

Line 366: Consider mentioning whether any efforts were made to minimize data entry errors during the transfer of paper forms to REDCap.

*Thank you, we added additional information in the limitations.*

Line 375: The conclusion mentions the need for public health education campaigns. Could you suggest specific strategies for these campaigns, such as which organizations might be responsible or which media could be used effectively?

*Thank you for raising this, we have made changes to the Conclusion.*

**Reviewer #3**:

The authors adequately explain the importance of the article within its field.

*Thank you, we appreciate your kind words.*

The methodology is sound, even considering the multiple sampling strategies due to logistics.

Considerations:

Lines 358 to 360: The survey was conducted with the head of household. Culturally, in Northern Tanzania, is the head of household the medical decision maker? For example, in Togo, head of household is often a male but decisions regarding medical need for any family members is managed by the household manager, usually the mother or another older woman. Could a mismatch be a limitation in this study? (Lines 154-155: In methods, informed consent was from both HoH and health care decision maker, but it is not clear if the health care decisionmaker performed the interview)

*Thank you. In Tanzania, the head of household is often also the healthcare decision-maker. There is a possibility of a mismatch, however, we feel confident based on knowledge of existing cultural practices, that the person interviewed is the healthcare decision-maker.*

Line 359: Responded “to”? Or responded “for”?

*This was corrected. Thank you.*

Line 370: The actual conclusion of the study is not listed in the conclusion section of the manuscript (though it can be seen in the abstract). The next steps listed are appropriate and highly relevant.

*Thank you for this. We have added a line to the Conclusion section.*

Lines 364 to 365: Are there other studies (likely outside the direct emergency medicine sector) in Tanzania or surrounding areas, that have investigated the understanding of emergencies? The language or culture of an emergency, perhaps anthropologically? Though this could be limited, such information could add context and depth for this and future research.

*We appreciate and agree with this important suggestion. We have included a recent reference from Uganda which assessed a similar question (lines 430-431). A larger discussion on this is probably not relevant to this manuscript, but would be an interesting future study for this region.*

References - 60% of the supporting articles were published in the past 7 years with many studies from geographically proximal areas which improves relevance.
